# Spatial relation learning in complementary scenarios with deep neural networks

**DOI:** 10.3389/fnbot.2022.844753

**Published:** 2022-07-28

**Authors:** Jae Hee Lee, Yuan Yao, Ozan Özdemir, Mengdi Li, Cornelius Weber, Zhiyuan Liu, Stefan Wermter

**Affiliations:** ^1^Knowledge Technology Group, Department of Informatics, University of Hamburg, Hamburg, Germany; ^2^Natural Language Processing Lab, Department of Computer Science and Technology, Tsinghua University, Beijing, China

**Keywords:** spatial relation learning, deep neural networks, hybrid architecture, embodied language learning, distant supervision, frame of reference

## Abstract

A cognitive agent performing in the real world needs to learn relevant concepts about its environment (e.g., objects, color, and shapes) and react accordingly. In addition to learning the concepts, it needs to learn *relations* between the concepts, in particular spatial relations between objects. In this paper, we propose three approaches that allow a cognitive agent to learn spatial relations. First, using an embodied model, the agent learns to reach toward an object based on simple instructions involving left-right relations. Since the level of realism and its complexity does not permit large-scale and diverse experiences in this approach, we devise as a second approach a simple visual dataset for geometric feature learning and show that recent reasoning models can learn directional relations in different frames of reference. Yet, embodied and simple simulation approaches together still do not provide sufficient experiences. To close this gap, we thirdly propose utilizing knowledge bases for disembodied spatial relation reasoning. Since the three approaches (i.e., embodied learning, learning from simple visual data, and use of knowledge bases) are complementary, we conceptualize a cognitive architecture that combines these approaches in the context of spatial relation learning.

## 1. Introduction

Spatial concepts and relations are essential for agents perceiving and acting in the physical space. Because of the ubiquitous nature of spatial concepts and relations, it is plausible from a developmental point of view to believe that they are “among the first to be formed in natural cognitive agents” (Freksa, 2004). Endowing an artificial cognitive agent with the capability to reliably handle spatial concepts and relations can thus be regarded as an important task in AI and, in particular, in deep learning, which has become a predominant paradigm in AI (LeCun et al., 2015).

In this paper, we present three different but complementary approaches to spatial relation learning with deep neural networks and propose a way to integrate them. In the first approach, a robotic agent collects experiences in its environment, learning about space in an *embodied* way. This approach allows the agent to ground the embodied experience similar to how humans would do and help the agent learn more accurate linguistic concepts suitable for human-robot interaction (Bisk et al., 2020). In such a learning setup, however, the variety of experiences is limited due to multiple factors such as exploration costs, limited complexity of explored environments, robot limitations in sensory, processing, and physical capabilities, which are not only present in the real physical environments, but also to a lesser extent in simulated environments. To increase the variety of experiences, further approaches are required.

The second approach utilizes computer-generated large-scale *image data* for spatial relation learning. An immediate advantage of this approach is that data generation is cheaper than in the first approach, such that training a large model with millions of samples is possible. This allows learning of complex relations, which can depend on different frames of reference. However, concerning detail, the simplified sensory input is insufficient for embodied multimodal learning. Furthermore, concerning variety, the number of automatically generated relations is still not on par with the variety of relations encountered in the real world.

In the third approach, a diverse and large amount of structured *data from knowledge bases* is used, which can be manually curated, crowd-sourced, or extracted from text resources on the web. This kind of data reflects human knowledge and experiences in unlimited domains beyond any specific scenarios. A limitation of this approach is that it accesses primarily semantic information from which spatial relations need to be inferred^1^ and they often do not involve directional relations^2^.

**Figure 1:**
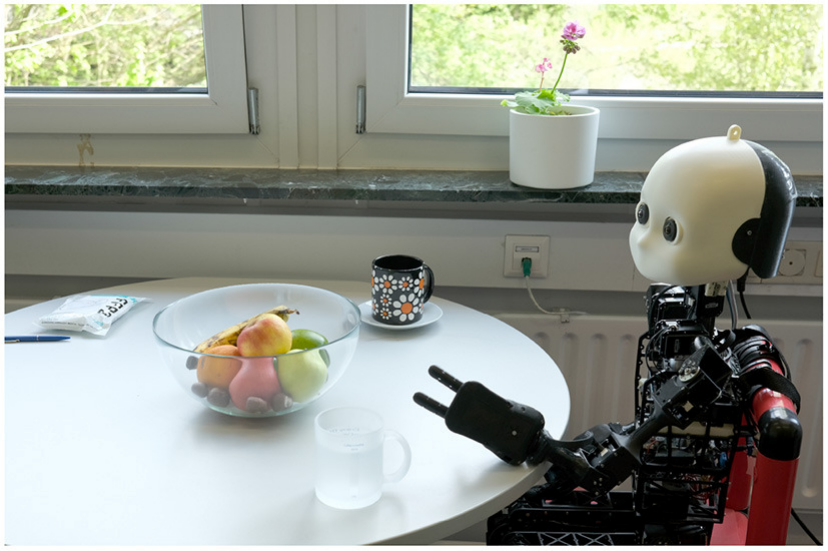
Spatial relations between objects can be obtained in different ways. Consider the instruction to the robot: “Take the cup to the left of the fruit bowl to water the plant.” Prior embodied experiences are needed for grounding the instruction in the real world. From its camera image, the robot can infer that there are cups on the table, but it needs to resolve “to the left of the fruit bowl” to use the correct cup. To infer that the plant, which is not in the robot's field of view, is on the windowsill, the robot can use prior knowledge, e.g., retrieved from a knowledge base, since it is a typical location for a plant.

These different, complementary approaches of data access have fostered the development of distinct tasks and of distinct classes of models: typical *embodied* models process sequences of multimodal data and output actions for robot control; models using disembodied *image data* are frequently used for classification; models using disembodied *data from knowledge bases* process symbolic information and are often used for inference and reasoning. We argue that all three approaches, although not directly compatible, are necessary to solve real-world tasks that involve spatial relation learning (cf. Figure 1). In this paper, we provide an example model for each of the three approaches. Moreover, we sketch a concept for their integration into a unified neural architecture for spatial relation learning.

Our contributions in this paper can be summarized as follows:

1. We test an *embodied* language learning model on a realistic scenario with a 3D dataset including spatial relations (Section 3).
2. We present a new *image data* set and evaluate state-of-the-art models on spatial relation learning (Section 4).
3. We propose a way to apply a relation learning approach that uses *data from knowledge bases* to learning spatial relations (Section 5).
4. We provide a concept for integrating the three approaches and discuss further extension possibilities (Section 6).

## 2. Related work

In this section, we discuss previous work that is relevant for this paper, where we discuss models for spatial relation learning and embodied language learning. We introduce datasets that involve spatial relations and contrast them with the *Qualitative Directional Relation Learning* (QDRL) dataset that we propose in this paper.

### 2.1. Embodied learning models

For embodied learning, an embodied agent (e.g., a robot) needs to act using its whole body or parts thereof (e.g., arms, hands, etc.) in an environment. As we are interested in spatial relation learning within the scope of this paper, and since it is a subset of language learning, in this part, we refer to the models that learn language in an embodied fashion. Specifically, we focus on embodied language learning with object manipulation. For a detailed and extensive review on language and robots, please refer to Tellex et al. (2020) where NLP-based robotic learning approaches are compared and categorized based on their technicalities and the problems that they address.

Early robotic language learning approaches focused on mapping human language input to formal language which could be interpreted by a robot (Dzifcak et al., 2009; Kollar et al., 2010; Matuszek et al., 2012, 2013). Dzifcak et al. (2009) introduced an integrated robotic architecture that parsed natural language directions created from a limited vocabulary in order to execute actions and achieve goals in an office environment by using formal logic. Similarly, Kollar et al. (2010) proposed an embodied spatial learning system that learned how to navigate in a building according to given human language input by mapping the natural language directions into formal language clauses and grounding them in the environment to find the most probable paths. Further, Matuszek et al. (2013) introduced an approach that could parse natural language commands to a formal robot control language (RCL) in order to map directions to executable sequences of actions depending on the world state in a navigation setup. Moreover, Matuszek et al. (2012) put forward a joint multimodal approach that flexibly learned novel grounded object attributes in the scene based on the linguistic and visual input using an online probabilistic learning algorithm. These early works were all symbolic learning approaches, while we are interested in neural network-based learning approaches in this paper.

As embodied language learning usually involves executing actions according to language input or describing those actions using language, it generally requires not only language and visual perception but also proprioception. Recently, numerous studies have been reported in which different objects are manipulated by a robot for embodied language learning (Hatori et al., 2017; Shridhar and Hsu, 2018; Yamada et al., 2018; Heinrich et al., 2020; Shao et al., 2020). Hatori et al. (2017) present a multimodal neural architecture which is composed of object recognition and language processing modules intended to learn the mapping between object names and actual objects as well as their attributes such as color, texture, or size for moving them to different boxes, especially in cluttered settings. Shridhar and Hsu (2018) introduce the INGRESS (interactive visual grounding of referring expressions) approach that has two streams, namely self-reference (describing the object with inherent characteristics in isolation) and relation (describing the object according to its spatial relation to other objects), and that can generate language expressions from input images to be compared with input commands to locate objects in question to pick them with the robotic arm. Yamada et al. (2018) propose the paired recurrent autoencoders (PRAE) model, which fuses language and action modalities in the latent feature space *via* a shared loss, for bidirectional translation between predefined language descriptions and simple robotic manipulation actions on objects. Heinrich et al. (2020) propose a biologically inspired crossmodal neural network approach, the adaptive multiple timescale recurrent neural network (adaptive MTRNN), which enables the robot to acquire language by listening to commands while interacting with objects in a playground environment. Shao et al. (2020) put forward a robot learning framework that combines a neural network with reinforcement learning, which accepts a linguistic instruction and a scene image as input and produces a motion trajectory, trained to obtain concepts of manipulation by watching video demonstrations from humans.

### 2.2. Spatial relation learning datasets

Spatial relation learning can be understood as a subproblem of visual relationship detection (VRD) (Lu et al., 2016; Krishna et al., 2017) that has as its task predicting the subject-predicate-object (SPO) triples from images. As the SPO triples are often biased toward frequent scenarios (e.g., a book on a table), datasets such as the UnRel Dataset (Peyre et al., 2017) and the SpatialSense dataset (Yang K. et al., 2019) were proposed to reduce the effect of the dataset bias. A task that is more general than visual relation detection and implicitly requires spatial relation learning is visual question answering (VQA), whose goal is to answer questions on a given image (Antol et al., 2015; Goyal et al., 2017; Wu et al., 2017).

Existing datasets for VRD and VQA do not distinguish between different frames of reference, which aggravates not only the difficulty of spatial relation prediction but also the difficulty of analyzing the performance of the models. To overcome the limitations of the existing datasets, in Section 4 we propose the *Qualitative Directional Relation Learning* (QDRL) dataset for analyzing the model performance on spatial relation learning in different frames of reference. Similar to the existing visual reasoning datasets CLEVR (Johnson et al., 2017), ShapeWorld (Kuhnle and Copestake, 2017), and SQOOP (Bahdanau et al., 2019), QDRL is a generated dataset that allows for controlled evaluations of the models. But different from the former three datasets, whose spatial relations are exclusively based on an *absolute* frame of reference, QDRL also allows us to test model performance concerning *intrinsic* and *relative* frames of reference.

### 2.3. Spatial relation learning models

One of the early approaches to learning spatial relations is the connectionist model proposed in Regier (1992), which was developed as a part of the L_0_ project (Feldman et al., 1996). As an early connectionist model it is characterized by its involvement of several hand-engineered components, e.g., the object boundaries and orientations of the objects are preprocessed and not learned from data. In Collell and Moens (2018), the authors propose a model that predicts the location and the size of an object based on another object that is in relation to it. The model uses bounding boxes and does not distinguish between left and right for location and size prediction. For general VRD and VQA problems, most models rely on the cues from the language models they employ and use the bounding box information (Wu et al., 2017; Lu et al., 2019; Tan and Bansal, 2019). Contrary to the VRD and VQA models, models for visual reasoning such as FiLM (Perez et al., 2018) and MAC (Hudson and Manning, 2018) do not rely on bounding boxes or pretrained language models. Furthermore, these two models do not assume any task-specific knowledge, which is for example exploited by neuro-symbolic approaches or neural module networks (Andreas et al., 2016; Yi et al., 2018).

## 3. Embodied spatial relation learning

Having a body and acting in the environment is essential for cognition as human cognition relies upon having embodied context-dependent sensorimotor action capabilities in the environment, i.e., perception and action are inseparable in experienced cognition (Varela et al., 2017), since humans perceive the world through a variety of sensors and act in the world with their motor functions (Arbib et al., 1986). Similarly, machines cannot truly infer true meanings from words without experiencing the real world with vision, touch, and other sensors (Arbib et al., 1986). Therefore, embodiment is also a necessary condition in spatial relation learning: by having an embodied agent situated in the environment we can learn grounded meanings of spatial relations such as left or right.

A simple robotic scenario generally involves a robot manipulating a few objects on a table. The robot may either execute actions according to given commands in textual/audio form or translate actions to commands. This requires a crossmodal architecture that involves multiple modalities like vision, language, and proprioception. Using multiple modalities helps for the case of spatial relation learning since seeing the object to be manipulated (vision), grounding commands that are associated with actions (language) and registering joint angle trajectories (proprioception) are all different interpretations of the world. For example, when executing a command such as “push the left object,” both seeing the objects on the table and moving the arm of the robot in the correct trajectory with learned joint angles support learning the position “left.”

### 3.1. A bidirectional embodied model

**Figure 2:**
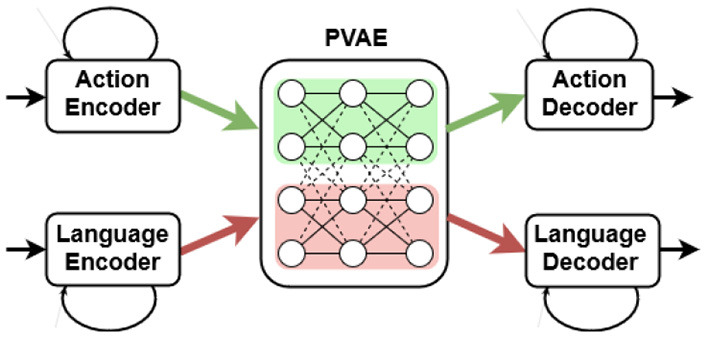
Bidirectional embodied model.

**Figure 3:**
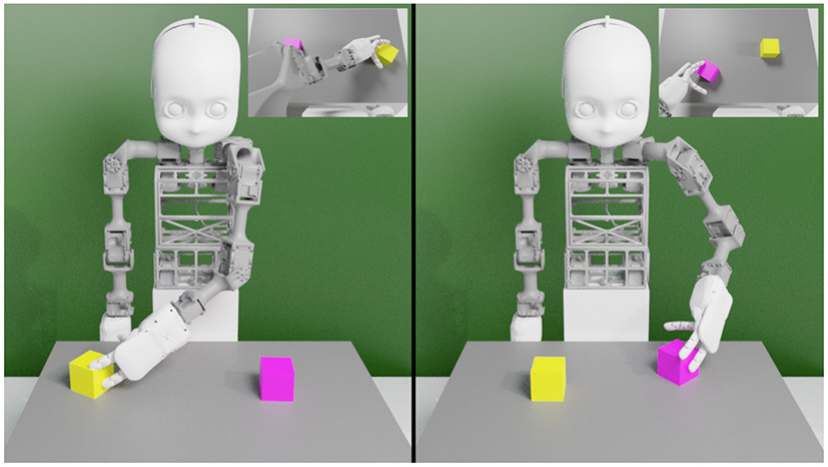
The NICO robot (Kerzel et al., 2017) in the simulation environment (Özdemir et al., 2021). **(Left)** NICO is sliding the right cube. **(Right)** NICO is pulling the left cube. In both segments, NICO's field of view is shown in the top right insets.

A bidirectional embodied model, such as the PRAE (paired recurrent autoencoders; Yamada et al., 2018), is attractive to approach grounding of language, since it is able to both execute simple robot actions given language descriptions and to generate language descriptions given executed and visually perceived actions. In our recent extension of the model in a robotic scenario (Özdemir et al., 2021), schematically shown in Figure 2, two cubes of different colors are placed on a table at which the NICO robot (Kerzel et al., 2017) is seated to interact with them (see Figure 3). Given proprioceptive and visual input, the approach is capable of translating robot actions to textual descriptions. The proposed Paired Variational Autoencoders (PVAE) extension allows to associate each robot action with eight description alternatives, and provides one-to-many mapping, by using Stochastic Gradient Variational Bayes (SGVB).

The model consists of two autoencoders: a language and an action VAE. The language VAE learns descriptions while the action VAE learns joint angle values conditioned on the visual input. After encoding, the encoded representations are used to extract latent representations by randomly sampling from a Gaussian distribution. A binding loss brings the two VAEs closer by reducing the distance between two latent variables. Additionally, we introduced a channel-separated CAE (convolutional autoencoder), for the PVAE approach, for extracting visual features from the egocentric scene images (Özdemir et al., 2021). The channel separation refers to training the same CAE once for each RGB channel and concatenating the features extracted from the middle layer of the CAE for each color channel to arrive at the combined visual features. The PVAE with channel-separated CAE visual feature extraction outperforms the standard PRAE (Yamada et al., 2018) in the one-to-many translation of actions into language commands. The approach is significantly more successful in the case of three color alternatives per cube and also with six color alternatives compared to PRAE. Our findings suggest that variational autoencoders facilitate better one-to-many action-to-description translation and address the linguistic ambiguity between an action and its probable descriptions in the simple scenario shown in Figure 3. Moreover, channel separation in visual feature extraction leads to a more accurate recognition of object colors.

### 3.2. Embodied spatial relation learning dataset

The previously mentioned works on bidirectional autoencoders do not experiment with the models' spatial relation learning capabilities. The instructions that the model processes are composed of three words with the first word indicating the type of action (push, pull, or slide), the second the cube color (six color alternatives) and the last the speed at which the action is performed (slowly or fast). The command “slide yellow slowly” and “pull pink fast” are example descriptions used for the model (cf. Figure 3). Therefore, the corpus includes 36 possible sentences (3 action × 6 color × 2 speed) without the alternative words and 288 possible sentences are created by replacing each word with an alternative (36 × 2^3^). Moreover, the dataset consists of 12 action types (e.g., push left, pull right etc.) and 12 cube arrangements (e.g., pink-yellow, red-green etc.), thus of 144 patterns (12 action type × 12 arrangement).

We extend this corpus by adding “left“ or “right“ as a new term to each description. Therefore, above example descriptions become “slide right yellow slowly” and “pull left pink slowly,” respectively—the descriptions are composed of four words.^3^ Color words may also be omitted so that the model needs to rely on the spatial specification. For simplicity, the cubes are placed on two fixed positions and the two cubes on the table are never of the same color. We have trained the model with the modified descriptions using the same hyperparameters as in Özdemir et al. (2021) for 15,000 iterations with a learning rate of 10^−4^ and batch size of 100^4^.

### 3.3. Results of the PVAE model

To translate actions to descriptions, we use the action encoder and language decoder: given joint angle values and visual features, we expect the model to produce the correct descriptions. For the bidirectional aspect of PVAE, we also test the language-to-action translation capability. For this task, we give as input one of the eight alternative descriptions for each pattern (action-description-arrangement combination) and we expect the model to predict the corresponding joint angle values. To that end, we use the language encoder and action decoder of PVAE. Both tasks are evaluated using the same trained model.

**Figure 4:**
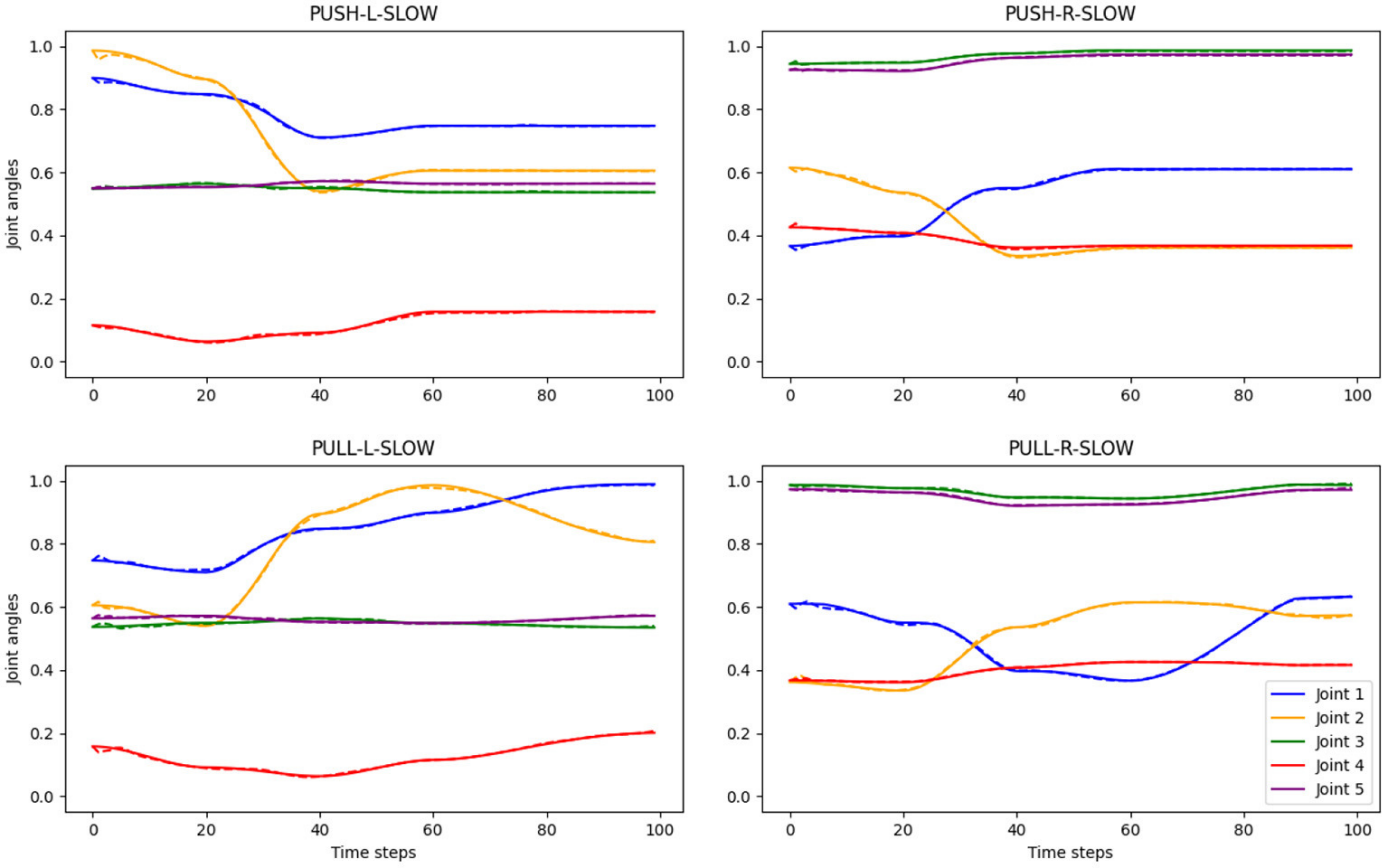
Examples of original and predicted joint angle trajectories for four different actions. The predicted values are generated by PVAE, given language descriptions and conditioned on visual input. Solid lines show the ground truth, while the dashed lines, which are often hidden by the solid lines, show the predicted joint angle values. The titles denote the action types, e.g., “PULL-R-SLOW” means pulling the right object slowly. The ground truth action trajectories with joint angle values were generated with an inverse kinematics solver in the simulation environment (Özdemir et al., 2021).

The results are as follows:

- PVAE is able to translate from actions to descriptions with 100% accuracy for all 144 patterns, including 108 training and 36 test patterns (see Table 1). This matches the results reported in Özdemir et al. (2021).
- The predicted joint angle values are tightly close to the original values, as can be seen in Figure 4 with qualitative results and in Table 1 with average quantitative results in terms of the normalized root-mean-square error (nRMSE) between the original and predicted joint trajectories. Therefore, we expect the robot to execute correct actions according to the given instructions.

**Table 1:** Performance of PVAE on bidirectional translation.

**Translation type**	**Evaluation measure**	**Train (%)**	**Test (%)**
Action → Language	Description accuracy↑	100	100
Language → Action	nRMSE↓	0.53	0.55

Green background indicates good performance.

It is arbitrary to describe an action with both the relative position and color of the object being manipulated in this scenario due to the cube arrangements. However, when two cubes of the same color are present on the table, adding relative position information into descriptions is necessary to avoid confusion—we do not test this since the dataset (Özdemir et al., 2021) does not involve two cubes of the same color simultaneously on the table. Furthermore, we can also be sure that the same action-to-description translation performance could be achieved by removing the color term from the descriptions as the position information of the object can be extracted through proprioception only, i.e., joint angle values, without the need for the vision modality. This is because the position of the cube being handled can be inferred from the proprioception as action types include the position (left or right) rather than the color of the cube.

For practical reasons, we do not simulate the robot with predicted joint angle trajectories. Due to certain subtleties in object manipulation (contact point etc.), there may be divergences in the simulated kinematics where the objects are moved toward compared to original trajectories. Note further that, compared to a human of the same size, the arm movements of our robot (i.e., the NICO robot; Kerzel et al., 2017) are more constrained due to fewer degrees of freedom, short arms, self-obstruction by the limbs, and by its inflexible trunk. We, therefore, set up only a simple scenario with left-right relation in the robot's egocentric frame of reference. In the following section, we tackle more complex spatial problems with multiple frames of reference.

## 4. Spatial relation learning using image data

Investigating how well neural networks learn the geometric features underlying different spatial relations is an important step toward building robust deep learning models for learning spatial relations. In this section, we propose a new dataset that we call the *Qualitative Directional Relation Learning* (QDRL) dataset, which allows for testing the performance of deep learning models on directional relational learning tasks. We evaluate the performance of representative end-to-end neural models on the QDRL dataset concerning different frames of reference and their generalizability to unseen entity-relation combinations (also known as compositional generalizability).

### 4.1. Directional relation learning

Humans adopt different strategies when giving instructions to robots, where different frames of reference play a role (Tenbrink et al., 2002). There are three kinds of frames of reference according to Levinson (1996). In an *absolute* frame of reference, the location of an entity is given by a fixed frame of reference shared by all entities (cf. **Figure 7**). In an *intrinsic* frame of reference, each object determines the reference frame given by its orientation (cf. **Figure 7**). In a *relative* frame of reference, the direction between two entities determines the frame of reference for locating another third entity (cf. **Figure 7**).

**Figure 5:**
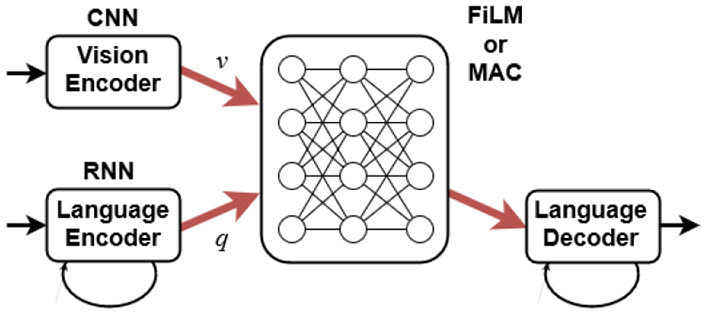
Spatial relation learning model.

In this section, we evaluate two deep learning models, FiLM (Perez et al., 2018) and MAC (Hudson and Manning, 2018). Schematically, Figure 5 shows that they take as input a raw RGB image and a question as a sequence of strings. These are turned into vectors *v* and *q* using a convolutional neural network (CNN) and a recurrent neural network (RNN), respectively. They produce a text answer as output, which here reduces to true or false. The two models differ in how they process *v* and *q*. As generic visual reasoning models they are fully differentiable and do not assume any task-specific knowledge (e.g., bounding boxes or the structure of the question).

The FiLM network processes *v* through a sequence of ResNet (He et al., 2016) blocks where the output values before the final ReLu activation function in each block are affinely transformed. The parameters for the affine transformations are obtained from the question vector *q* through a linear transformation. This way, FiLM allows the question to modulate what information passes through each ResNet block function, which helps sequential reasoning.

The main idea of the MAC network is to model a reasoning process by keeping a sequence of control operations and a recurrent memory, where the control operations decide what information to retrieve from the image, and the memory retains information relevant for each reasoning step.

### 4.2. The qualitative directional relation learning dataset

**Figure 6:**
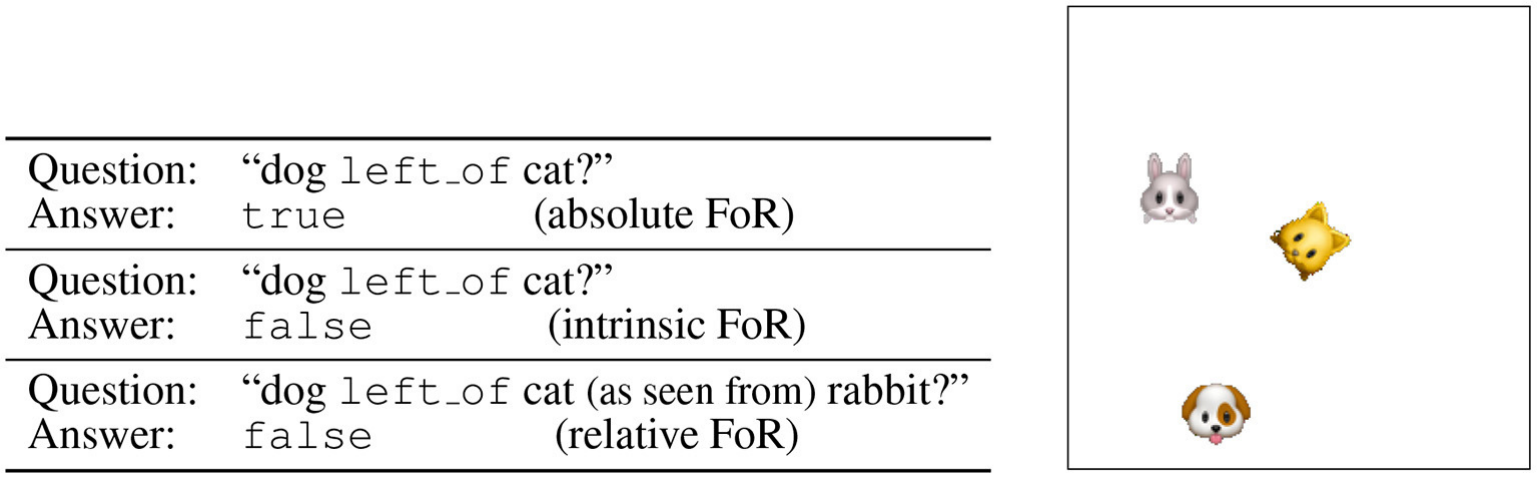
An example of a QDRL dataset sample. Given an image, in an absolute and an intrinsic frame of reference a question about the image is a triple (entity1, relation, entity2), and in a relative frame of reference, a question about the image is a quadruple (entity1, relation, entity2, entity3). As can be seen in the ground truth answers to the question, different frames of reference (FoR) lead to different answers.

The Qualitative Directional Relation Learning (QDRL) dataset we propose consists of (image, question, answer) triples^5^. Here, the *question* is a simple statement about the spatial relation between the objects in the image and is of the form (*head, relation, tail*), e.g., (rabbit, left_of, cat). The *answer* can be either true or false and depends on the adopted frame of reference, and the distribution of the truth values of the answers is balanced, such that no bias can be exploited. The *image* is of size 128 × 128 with a black background and contains non-overlapping entities of size 24 × 24. As entities we chose face emojis that have clear front sides and facilitate detecting orientations. The samples are generated as follows. First, a fixed number *n* of emoji names are randomly chosen from 38 possible emoji names. Then a head entity *h*, a tail entity *t*, a relation *r* and an answer *a* are randomly selected so as to form an (*h, r, t*) question triple and the ground-truth answer *a*. To prepare a corresponding image, the *n* entities are randomly rotated and placed in the image until the constraint [(*h, r, t*), *a*] is satisfied. An example with ground truth answers concerning different frames of reference is given in Figure 6.

**Figure 7:**
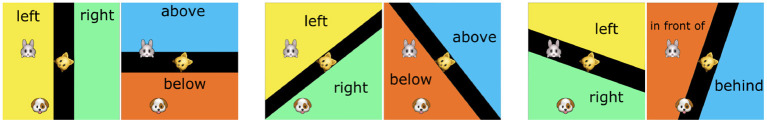
The three frames of reference according to Levinson (1996). **(Left)** In an *absolute frame of reference*, the location of an entity is given by a fixed frame of reference shared by all entities (above is fixed to the north, here, of the cat). **(Middle)** In an *intrinsic frame of reference*, an object has its own frame of reference given by its orientation (here, the cat is oriented toward the northeast). **(Right)** In a *relative frame of reference*, the direction given by two entities (here, the direction from the rabbit to the cat) determines the frame of reference for locating another third entity (here, dog).

As directional relations we use {above, below, left_of, right_of} for absolute and intrinsic frames of reference and {in_front_of, behind, left_of, right_of} for a relative frame of reference. Examples of the directional relations, where frames of reference are taken into consideration, are given in Figure 7.

The QDRL dataset encourages a neural network model to learn the (oriented) bounding box of the reference entity as it induces the decision boundaries for different relations, where the kind of bounding box a model has to learn depends on the given frame of reference. In an absolute frame of reference, a model has to learn the axis-aligned bounding box of the reference entity. In an intrinsic frame of reference, a model has to additionally learn the orientation of the reference entity and the bounding box that is aligned to that orientation. In a relative frame bsof reference, a model has to determine the centers of the reference entity and the source entity that “sees” the reference entity and align the bounding box to the direction from the center of the source entity to the center of the reference entity.

### 4.3. Experiments on the QDRL dataset

In this section, we evaluate the performance of the FiLM and the MAC networks on the QDRL dataset with respect to different frames of reference and their compositional generalizability, i.e., their generalizability to unseen entity-relation combinations. To this end, we train the two models on 1,000,000 (image, question, answer) triples and validate on 10 10,000 (image, question, answer)-triples, where we vary the following parameters for each experiment: (i) frame of reference and (ii) for absolute and intrinsic frames of reference the number of entities in each scene (∈{2, 5}).

In addition to the standard validation set, to test how the models generalize compositionally, we hold out a subset *S* of 18 entities from the 32 entities appearing in the training set and make sure that every question in the training set involves at least an entity that is not in *S*. We then create a dataset consisting of 10,000 (image, question, answer) triples exclusively with the entities from *S* and call it the *compositional validation set*. This way it is guaranteed that the set of questions in the training set has no overlap with the set of the questions in the compositional validation set. This allows us to test whether a model is able to learn to disentangle entities and relations as well as to learn the syntactic structures, such that they can deal with unseen combinations of entities and relations.

All model hyperparameters, except for the number of FiLM blocks (∈{2, 4, 6}) and the MAC cells (∈{2, 8}) that we optimize, are taken from Bahdanau et al. (2019). For training, we choose 32 as the batch size and apply early stopping based on the model's performance on the validation set.

### 4.4. Results by FiLM and MAC models

In Table 2, we report the accuracy results of the experiments. From the table, we can observe the following.

- Learning directional relations in an intrinsic frame of reference and a relative frame of reference is more challenging than in an absolute reference, which intuitively makes sense as the models have the extra burden to learn the orientations.
- All models achieve relatively high performance on the validation set, which indicates that both FiLM and MAC have sufficient capacity to learn the training distribution.
- Regarding the compositional generalization set, for the FiLM model the difficulty of the tasks increases in the order of absolute, intrinsic, and relative frame of reference, whereas the MAC model is not affected by the frames of reference, and consistently outperforms the FiLM model. The performance gap between MAC and FiLM is significant in the case of relative frames of reference.
- The MAC model shows overall a smaller gap between the performances on the validation set and the compositional validation set. Even though MAC does not perform better than FiLM on the validation set, its performances on the compositional validation set are consistently better than those of FiLM.

**Table 2:** Accuracies of FiLM and MAC networks on the QDRL dataset.

**FoR** ^*a*^	# **Ents**^*b*^	**FiLM**		**MAC**	
		**Val** ^*c*^	**Comp** ^*d*^	**Val**	**Comp**
Absolute	2	0.996	0.912	0.985	0.929
	5	0.996	0.933	0.992	0.958
Intrinsic	2	0.979	0.882	0.973	0.927
	5	0.978	0.862	0.967	0.937
Relative	3	0.978	0.745	0.978	0.975

a Frame of reference.

b # Entities.

c Validation set.

d Compositional validation set. Green background indicates good performance, red indicates worse performance.

These results demonstrate the good ability of neural networks to learn spatial relations in diverse frames of reference. However, due to the simplicity of the simulated dataset, it will be necessary to test the models' capabilities on more realistic 3D data (cf. Section 3). Since it is difficult to model the prior knowledge about spatial relations in the real world, in the following section we will consider the possibility of making use of existing knowledge bases.

## 5. Spatial relation learning using knowledge bases

People have created several large-scale commonsense knowledge bases to store relational knowledge about objects in structured triples (Speer et al., 2017; Ji et al., 2021; Nayak et al., 2021), such as (person, riding, horse) and (plant, on, windowsill). Intuitively, relational triples in commonsense knowledge bases store expected prior relations between objects, which can provide useful disembodied learning signals for relation detectors. Combined with object detectors, the relation detectors can produce structured graph representations of the scene, which can be useful for robots to obtain a deep understanding of the environment and perform subsequent interactions.

**Figure 8:**
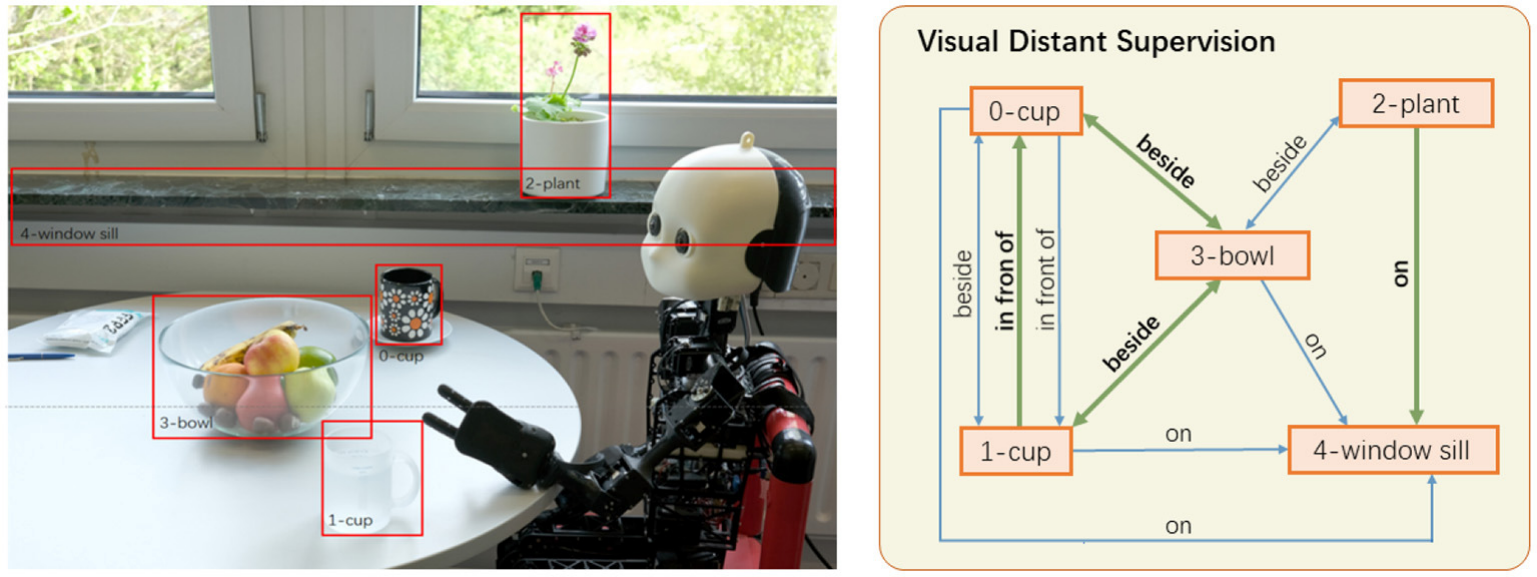
Visual distant supervision (Yao et al., 2021) retrieves plausible relations between the detected objects (only a selection of bounding boxes and relations is shown). Correct relation labels are highlighted in bold and green thick arrows.

To leverage commonsense knowledge bases for visual relation detection, we have proposed the visual distant supervision technique in Yao et al. (2021). Visual distant supervision aligns commonsense knowledge bases with unlabeled images to automatically create distantly labeled relation data, which can be used to train any visual relation detectors. The underlying assumption is that the relations between two objects in an image tend to be the same as their relations in the knowledge bases. As shown in the example in Figure 8, since the object pair (bowl, cup) is labeled with relation beside in knowledge bases, an image with object pair (bowl, cup) will have beside as a candidate relation for the pair. In this way, visual distant supervision can train visual relation detectors without any human-labeled relation data, achieving strong performance compared to semi-supervised relation detectors that utilize several seed human annotations for each relation (Chen et al., 2019).

However, the assumption of distant supervision inevitably introduces noise in its automatic label generation, such as the relation label beside for the object pair (bowl, plant) in Figure 8. The reason is that distant supervision only depends on object categories for relation label generation, without considering the complete image content or spatial layout. To alleviate the noise in distant supervision, we have proposed a denoising framework that iteratively refines the probabilistic relation labels based on the EM optimization method (Yao et al., 2021). When human-labeled relation data is available, pretraining on distantly labeled data can also bring in improvements over fully supervised relation detectors.

Despite its effectiveness in learning relations, distant supervision is not always useful for spatial relation learning (e.g., there is no prior knowledge about whether a cup with water should be to the left or to the right of the fruit bowl in Figure 8). However, some relations have implicit spatial information, which can potentially be useful for spatial relation learning. For example, the relation riding implies the spatial relation on, where this implication can be obtained from linguistic knowledge bases, such as WordNet (Fellbaum, 1998). Based on the implications, relation representations learned *via* distant supervision can be transferred to help spatial relation learning. Effectively leveraging distant supervision for spatial relation learning is, therefore, an important research problem.

## 6. Concept of an integrated architecture

The previous sections presented complementary models for spatial reasoning: a model to collect embodied, but costly, experiences; a model for plentiful, but oversimplifying, simulations; and a knowledge base enriched, but disembodied, technique. To achieve the intelligent behavior of an AI agent, the merits of such models must be combined. However, neural models mostly cannot be trivially combined by using a modular setup with well-defined interfaces. Since our models have overlapping functionality, their combination needs to be designed in the architecture and by joint training of the architecture components. In the area of multi-task learning, there have been recent attempts to tackle multiple datasets and tasks, combining multiple inputs and outputs, by a single model (Kaiser et al., 2017; Pramanik et al., 2019; Lu et al., 2020). The conjecture is that while multiple tasks are concurrently learned, learning one task can help the others. To transfer knowledge or skills, parts of the neural architecture are shared between the tasks.

**Figure 9:**
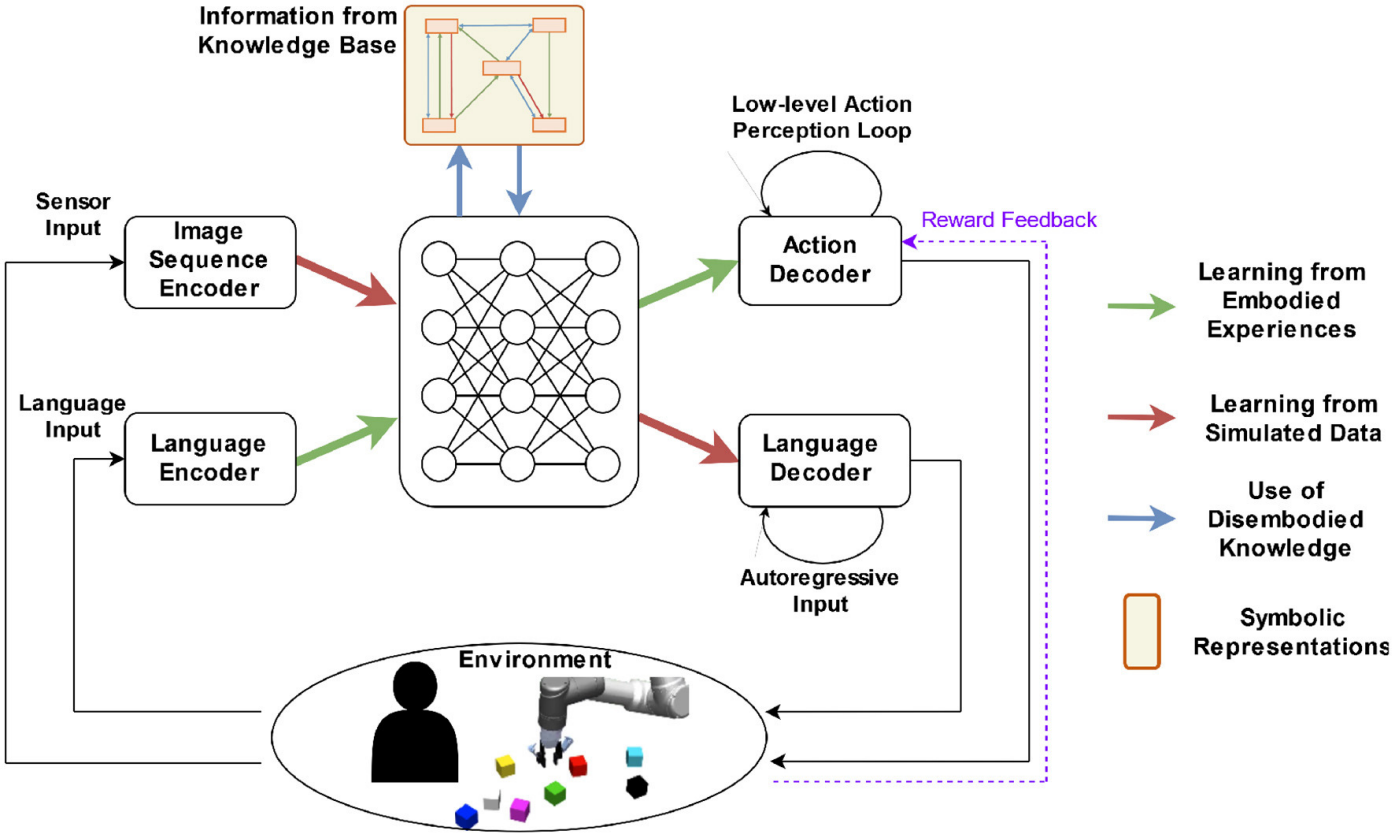
Concept of an integrated architecture for spatial relationship learning. Our presented models cover only input-to-output. The loop closure with the environment depicted here indicates an extension, such as dialogue with a human. Smaller loops on the decoders indicate low-level feedback-driven behaviors such as reaching a target object or producing a sentence.

Figure 9 shows a concept for our proposition that follows a bidirectional model architecture (cf. Section 3), which enables tasks in two directions: The task to act, given language instructions, is best performed by embodied learning in a realistic 3D simulation (green arrows indicate the direction of the information flow). The task to produce language descriptions, given (visual) sensor input (red pathway), lends itself to using simulated visual data containing geometric relations that can be easily produced in large quantities (cf. Section 4). The representations on the central part benefit from joint training by forming a joint abstract representation of entities, which are independent of the input modality. The bidirectionality of the model ensures compatibility with both directions, while a large overlap in the joint central part should ensure that extensive spatial relation learning on large datasets can help the other tasks.

Disembodied knowledge (cf. Section 5), e.g., from a knowledge base, enters the model as another input (blue arrows in Figure 9). The recognition of concepts, given language and sensor input, will activate related disembodied knowledge to help to finish the task, which can be achieved by first retrieving related relational triples from the knowledge base, and then obtaining enhanced representations of the language and sensor input with the retrieved knowledge. For example, when a robot is asked to “fetch the cup,” related relational triples will be retrieved, such as (cup, on, table), represented into embeddings, and integrated into the representation of the instruction so that the robot will expect to find the cup on the table first (see also Figure 1).

A practical methodology to incorporate disembodied knowledge into an integrated model is using a graph neural network (GNN) (Gori et al., 2005; Liu and Zhou, 2020). The disembodied knowledge is represented in the form of a graph structure where nodes capture the concepts and edges capture the existing relations between the nodes. Nodes hold a vector, while interactions over the edges are represented as neural networks, which share weights in case of same relation types. To compute a target function, vectors on each node are iteratively updated. The structure of the GNN can be derived from knowledge bases such as ConceptNet or Visual Genome, and the structure will be typically sparse, i.e., only a small proportion of node pairs will be connected. The trainable GNN parameters, including the input and readout connections that connect the GNN layer to the main neural model architecture (blue arrows in Figure 9), can be trained as part of the integrated architecture. This requires a dataset, in which the model function can benefit from the GNN, such as commonsense reasoning (Talmor et al., 2019). In our integrated model, the graph neural network would first need relevant nodes activated, which correspond to items from the visual input or to words from the language input. Such a mapping could be established by supervised pretraining. The GNN converts commonsense knowledge regarding object-to-object relations, which is encoded in its structure, to be used by the distributed representations of our neural model. Thereby the external knowledge gets fused with representations obtained from the instruction and visual signals in order to enhance spatial reasoning (Yang J. et al., 2019).

While our models are trained in a supervised way from pairs of input and output vectors, interacting with the environment means that actions are iteratively performed by an embodied learning agent (Figure 9 shows the environment in the loop). There are many approaches to train the action policy of an agent, including supervised learning (Shah et al., 2021), imitation learning (Chevalier-Boisvert et al., 2019; Chaplot et al., 2020; Shridhar et al., 2021), and reinforcement learning (Hermann et al., 2017; Chaplot et al., 2018; Li et al., 2021). Among these approaches, reinforcement learning is most versatile because it does not require human-labeled data for all situations, but the agent can learn its action policy by interacting with the environment and only occasionally receiving rewards (purple dashed arrow in Figure 9). The reward function is typically designed manually based on the domain knowledge of the target task, or it can be an intrinsic reward function (Pathak et al., 2017).

## 7. Discussion

### 7.1. Integrating reinforcement learning

Our bidirectional model is trained in a supervised fashion to perform physical actions in a continuous 3D space (Section 3). However, small deviations from a teacher trajectory could lead to failure, for example, in grasping an object. Reinforcement learning (RL), in contrast, is sensitive to the narrow regions in action space that distinguish successful from non-successful actions. In Figure 9, we therefore suggest using RL as a superior method for the physical actions.

Goal-conditioned RL is advisable for cases where the agent's goal not only depends on the state of the environment, but where the goal is also conditioned on further input, such as its internal state (Dickinson and Balleine, 1994), or on language input as in our model. Goal-conditioned RL furthermore underlies hierarchical RL, where a higher-level module dynamically sets goals for a lower-level module, and hindsight experience replay (HER), where a future state in any trajectory is set as a goal in hindsight. With the availability of abundant high-quality trajectories, Lynch and Sermanet (2021) use an imitation learning approach, where the agent uses HER to learn from crowd-sourced trajectories, where the goal representation is paired with language input, in order to realize a flexible language-to-action mapping.

While RL is established for learning physical actions and suitable for general use (Silver et al., 2021), its use for language learning is yet emergent (Röder et al., 2021; Uc-Cetina et al., 2021). Regarding language as a sequence production problem, our language decoder could benefit from the availability of high-quality forward models, such as the Transformer language model. Such a language model could be used as a forward model in a model-based RL algorithm, as done by the decision transformer (Chen et al., 2021) and the trajectory transformer (Janner et al., 2021). However, such open-domain language models are difficult to use in a visual context to achieve specified goals. In order to define terminal goals in RL for language learning in specific domains, simple visual guessing game scenarios were devised (Das et al., 2017; Zhao et al., 2021). The generated language can be further augmented for high-quality dialogue by rewarding certain properties like informativity, coherence, and ease of answering (Li et al., 2016), which works in open domains, or by other scores (e.g., BLEU or ROUGE) that compare to human-generated text (Keneshloo et al., 2020). The contrast between domain-specific scenarios, which allow to guide RL language learning *via* rewards, and open-domain sophisticated language models, reflects the contrast between embodied and simulated learning, which allows control over spatial relations, and the use of knowledge bases with their open-domain information.

A challenge for deep RL is that its many parameters are trained from sparse and often binary reward feedback. Therefore, unsupervised or supervised pretraining of model components, such as for sensory preprocessing, or for end-to-end components as described in Sections 3 and 4, can render deep RL efficient.

### 7.2. Curriculum learning

For machine learning tasks that span multiple levels of difficulty, curriculum learning has been shown to be efficient for a variety of models (Elman, 1993; Bengio et al., 2009).

In one of our experiments on the QDRL dataset (cf. Section 4) we observed that pretraining the FiLM model first on scenes in an *intrinsic* frame of reference with two objects and then fine-tuning it on scenes in a *relative* frame of reference with three objects helped the model to achieve about 0.9 accuracy on the compositional validation set, instead of 0.745 accuracy without the pretraining (cf. Table 2). This large increase in performance could be attributed to the fact that scenes in an intrinsic frame of reference are easier to learn as the relations involve only two objects, while at the same time helping a model to learn the concept of orientation. Fine-tuning on scenes in a relative frame of reference thus requires only modifying the concept of orientation, i.e., orientation is determined by two objects instead of intrinsically (cf. Figure 7).

A specific spatial relation between objects arises when one object occludes another, i.e., when one object is behind another from the observer's point of view. In the task of robotic object existence prediction by occlusion reasoning (Li et al., 2021), a robot needs to reason whether a target object is possibly occluded by a visible object. Curriculum learning has proven essential for the successful training of the proposed model. We found that training the model from scratch on data containing all types of scenes is hard. In the curriculum training strategy, the model is sequentially trained on four types of scenes with increasing difficulty. First, the model is trained on scenes with only one object. Then the model is trained on scenes with two objects but all of them are visible. Next, the model is trained on scenes with two objects with occlusion. In the end, the model is trained jointly on all possible scenes. After the curriculum learning, the obtained model is able to tackle all types of scenes well. Curriculum learning has also been proven useful in other works on embodied learning (Wu et al., 2019; Yang W. et al., 2019).

Models that use knowledge graphs can also benefit from gradually increasing levels of difficulty. For example, to decompose the prediction of a complex scene graph, Mao et al. (2019) propose to first predict easy relations that models are confident with, and then better infer difficult relations based on the easy ones. Zhang et al. (2021) leverage relation hierarchies in knowledge bases, and propose to first learn the coarse-grained relations that are distant in relation hierarchies, and then distinguish the fine-grained relations that are nearby in relation hierarchies.

### 7.3. Architecture extension possibilities

The combined model concept considers recurrent networks such as LSTMs to be used as the action and language encoders and decoders, following the bidirectional embodied model architecture presented in this paper. Pretrained Transformer-based language models like BERT (Devlin et al., 2019) do not have language grounded in the environment because they are trained exclusively on textual data—they are unimodal, with no visual or sensorimotor information considered. However, spatial reasoning requires visual and/or sensorimotor perception to make sense of whether an object is to the left or right of another. Therefore, in order to make use of a pretrained language model *via* transfer learning, we leave adopting a BERT model as a language encoder/decoder and fine-tuning it as part of future work. Integrating a language model in this manner should endow our combined model with commonsense knowledge without having to lose its spatial reasoning capabilities.

Learning spatial relations requires reasoning about the frame of reference. In Section 4, the task was to learn spatial relations when frames of reference are given. A more challenging scenario would be when frames of reference are not given explicitly but need to be inferred. We often encounter this scenario in real-world conversations: some people tend to take the perspective of others, whereas some tend to use the egocentric perspective. This gives rise to ambiguities, which need to be resolved in a dialogue through questions and answers.

Existing works have demonstrated that commonsense knowledge graphs can effectively facilitate visual relation learning. However, knowledge graphs are typically introduced to train a relation predictor to produce scene graphs for downstream tasks. To leverage the symbol-based scene graphs in downstream tasks, graph embedding models are usually needed, which makes the overall procedure expensive and cumbersome. In the future, knowledge graphs can be directly integrated into the representations of pretrained vision-language models during pretraining, helping the models to better learn objects and their relations. The knowledge in pretrained vision-language models can then be readily used to serve downstream tasks through simple fine-tuning.

## 8. Conclusion

In this paper, we have investigated multiple approaches for spatial relation learning. We have shown that an embodied bidirectional model can generate physical actions from language descriptions and vice versa, involving simple left/right relations. We have then shown on a new simple visual dataset that recent visual reasoning models can learn spatial relations in multiple reference frames, with the MAC model outperforming the FiLM model. Since it is unrealistic for a robot to learn exhaustive world knowledge through interaction, or through simple visual datasets, we have considered using the relations from knowledge bases to infer likely spatial relations in a current scene. A practical limitation that has become apparent in our study is that different datasets are needed to learn complementary aspects of spatial reasoning, which hampers the development of a single joint model. This limitation may be overcome by developing more comprehensive datasets, or by devising integrated modular architectures. Finally, we have presented a concept of such an integrated architecture for combining the different models and tasks, which still requires implementation and validation in the future. We furthermore discussed their extension possibilities, which can serve as a basis for intelligent robots solving tasks in the real world that require spatial relation learning and reasoning.

## Data availability statement

The code for reproducing the results in Section 4 can be downloaded from https://github.com/knowledgetechnologyuhh/QDRL.

## Author contributions

JL, OÖ, YY, and ML developed, implemented, and evaluated the models. CW, ZL, and SW helped in writing and revising the paper. JL and YY collected the data. All authors contributed to the article and approved the submitted version.

## Funding

This work was jointly funded by the Natural Science Foundation of China (NSFC) and the German Research Foundation (DFG) in Project Crossmodal Learning, NSFC 62061136001/DFG TRR-169.

## Conflict of interest

The authors declare that the research was conducted in the absence of any commercial or financial relationships that could be construed as a potential conflict of interest.

## Publisher's note

All claims expressed in this article are solely those of the authors and do not necessarily represent those of their affiliated organizations, or those of the publisher, the editors and the reviewers. Any product that may be evaluated in this article, or claim that may be made by its manufacturer, is not guaranteed or endorsed by the publisher.

## Footnotes

^1^For example, “the cup holds a drink” has the implicit spatial meaning that the drink is inside the cup (cf. Collell et al., 2018).

^2^According to Collell and Moens (2018), the spatial relations left and right comprise only <0.1% of the well-known visual genome dataset (Krishna et al., 2017).

^3^The use of left and right can involve reference objects that are not explicitly mentioned. For example, “slide right yellow slowly” implies that there is a reference object in the scene (e.g., the pink cube in Figure 3) and the yellow object is—as seen by the agent—to the right of the reference object.

^4^For more details on the hyperparameters and dataset details please refer to Özdemir et al. (2021).

^5^The code for reproducing the results in this section can be downloaded from https://github.com/knowledgetechnologyuhh/QDRL.
